# Formulation Patents and Dermatology and Obviousness

**DOI:** 10.3390/pharmaceutics3040914

**Published:** 2011-11-21

**Authors:** Dan-Feng Mei, Josephine Liu, Michael A. Davitz

**Affiliations:** 1 Fordham University School of Law, 140 West 62^nd^ Street, New York, NY 10023, USA; E-Mail: dmei@avhlaw.com; 2 Axinn Veltrop & Harkrider LLP, 114 West 47^th^ Street, New York, NY 10036, USA; E-Mail: jl@avhlaw.com

**Keywords:** patents, formulation, obviousness

## Abstract

Most patents covering dermatologic products contain patent claims directed to the pharmaceutical formulation of the product. Such patents, known as formulation patents, are vulnerable to attacks based on the legal argument that the formulations covered are obvious over formulations already known prior to the filing of the patent application. Because obviousness is an important concept in patent law, recent court cases concerning obviousness and formulation patents were examined and discussed below. Courts have ruled that patent claims are obvious when features of the claimed formulation are found in the prior art, even if the features or characteristics of the formulation are not explicitly disclosed in the prior art. However, patentees have successfully overcome obviousness challenges where there were unexpected results or properties and/or the prior art taught away from the claimed invention.

## 1. Introduction

Most patents covering dermatologic products fall in the formulation category. However, practically speaking, what is a formulation patent? As the name implies, a formulation patent is a patent that seeks to cover the pharmaceutical formulation of an active pharmaceutical ingredient, *i.e.*, the unique combination of the active pharmaceutical ingredient with excipients that make up the dosage form that is administered to the patient. Common dosage forms include a capsule, tablet, spray, lotion, gel, foam and may vary in the manner in which the active pharmaceutical ingredient is released, e.g., have an immediate-release or extended-release profile.

There are numerous dermatology products available by prescription. The formulation patents covering one such product, clobetasol, serve to illustrate the diversity of patent claims relevant to such products. Clobetasol, a U.S. Class I corticosteroid, is one of the most potent steroids available on the market [1]. It can be administered to a patient as a spray, gel, cream, aerosol foam, lotion, ointment, shampoo, or solution [2]. Clobetasol has been available as a topical ointment as early as the mid-1980s [3]. Nevertheless, there are various non-expired formulation patents containing claims that may cover a clobetasol product. This fact is illustrated by an excerpt from a publication provided by the U.S. Food & Drug Administration titled “Approved Drug Products with Therapeutic Equivalence Evaluations” (commonly referred to as the “Orange Book”), which lists all approved drug products and patents that cover the product [4].

While the illustration above focuses on formulation patents, it is important to note that the potential scope of claim coverage extends well beyond just formulations containing the drug. For example, it is also possible to claim a new salt or polymorphic crystal form of the active pharmaceutical ingredient, a new combination of known active ingredients, a new disease indication, a new route of drug administration, a new dosage amount, a new delayed release form, a new degree of compound purity and a new method of improving bioavailability [5].

Given that formulation patents represent a cornerstone for patent protection in the dermatologic space, we will explore a key aspect of their strength and weakness, namely obviousness.

## 2. Obviousness

A patent reflects a trade-off between the inventors and the U.S. government. In cases where the U.S. government, more specifically the United States Patent & Trademark Office (“USPTO”), determines that the public will benefit from knowledge of an invention because the invention is novel and unobvious, the government grants patent protection for the invention in exchange for public disclosure of the invention [6]. The inventor receives the benefit of excluding others from practicing the patented invention; the public receives the benefit of information – the disclosure of the invention. This broad concept embodies some of the important requirements governing the patentability of an invention. For example:

- The invention must be novel–it is something the public did not know about before [7];
- The invention is nonobvious–the invention is not some minor variation of something that is already in the prior art nor is it a variation that someone working in that field could have easily come up with [8]; and
- The inventor fully describes the invention in a way that fully discloses the invention [9].

Patents are all susceptible to challenges of novelty, usefulness, and obviousness. Formulation patents, in particular, are vulnerable to obviousness attacks.

An invention is unpatentable “if the differences between the subject matter sought to be patented and the prior art are such that the subject matter as a whole would have been obvious at the time the invention was made to a person having ordinary skill in the art” [10]. Obviousness is a difficult legal concept. In fact, during arguments on obviousness before the U.S. Supreme Court, Justice Scalia, commented that the key test for obviousness, the teaching-suggestion-motivation test (“TSM test”), is “gobbledygook[,] [i]t really is, it's irrational” [11]. If the test for obviousness is “gobbledygook” for a Justice of the highest court in the country, how can we make rational sense of the problem? Obviousness, at its core, really deals with predictability and the failure of the prior art to teach or instruct the inventor about what will happen when she combines two ingredients. In the leading Supreme Court case on obviousness, *KSR International Co. v. Teleflex, Inc.* [12], which threw out the rigid application of the TSM test for obviousness, the Court said that a combination of familiar elements is likely to be obvious “when it does no more than yield predictable results” [12]. But “when [the] prior art teaches away from combining certain known elements, discovery of a successful means of combining them is more likely to be nonobvious” [12].

In light of *KSR*, the USPTO issued guidelines for patent examiners to use to evaluate obviousness [13]. The following rationales, the USPTO says, may be used to support a finding of obviousness [14]:

1. Combining prior art elements according to known methods to yield predictable results;
2. Simple substitution of one known element for another to obtain predictable results;
3. Use of a known technique to improve a similar device (method or product) in the same way;
4. Applying a known technique to a known device (method or product) ready for improvement to yield predictable results;
5. “Obvious to try”–choosing from a finite number of identified, predictable solutions, with a reasonable expectation of success;
6. Known work in one field of endeavor may prompt variations of it for use in either the same field or a different one based on design incentives or other market forces if the variations are predictable to one of ordinary skill in the art;
7. Some teaching, suggestion, or motivation in the prior art that would have led one of ordinary skill to modify the prior art reference or to combine prior art reference teachings to arrive at the claimed invention.

In contrast, clear evidence of failure in the past or a so-called teaching away from the claimed combination of elements, points the way to why a new formulation or product would be nonobvious.

## 3. Guidance from Case Law

Although patent law is based in statute and stems ultimately from the Constitution, which guarantees inventors patent protection for a limited period of time [15], in order to truly understand dynamically how obviousness works we need to look at how courts interpret the law. Because formulation patents represent a key aspect for dermatology products, we are going to examine the relevant cases stemming from patent litigation over the question of obviousness of formulation patent claims.

### 3.1. Claims Are Obvious when the Claimed Ranges Overlap with the Prior Art and There Are No Unexpected Properties of the New Formulation

In *Tyco v. Mutual*, the claim in U.S. Patent No. 5,211,954 (“the '954 patent”) directed to 7.5 mg of hypnotic drug crystalline temazepam was found to be obvious [16]. Specifically, the district court held that the claim was obvious over prior art because prior to the purported invention date of the patent (i) pharmaceutical formulations of temazepam were sold in 15 mg and 30 mg dosages for more than a year before the priority date of the '954 patent; (ii) a medical reference book directed physicians to use temazepam at the dosage between 5 mg and 15 mg for the treatment of insomnia in the elderly; and (iii) parties agreed that “physicians always seek to prescribe the lowest effective dose of any medication, particularly hypnotics, such as temazepam” [16]. When the dosage range of the prior art overlaps with the claimed range, the claims are first presumed to be obvious [16]. This presumption that the claims are obvious is rebuttable by showing either that the prior art taught away from the invention or by showing that there are new and unexpected results relative to the prior art [16]. Here, however, the court held that the prior art did not teach away from the 7.5 mg capsules and there were no unexpected results to rebut the presumption of obviousness [16]. Tyco, the patentee, pointed to one prior art publication that specified the effectiveness of higher doses of temazepam (20 mg) in increasing total sleep time and decreasing sleep onset latency but the court was not persuaded that the art taught away from the efficacy of the 7.5 mg capsules [16]. Furthermore, even if the prior art taught away from the use of 7.5 mg temazepam capsules generally, it would not cast doubt on the medical book reference that disclosed the recommended dosage for elderly patients to be 5 mg to 15 mg of temazepam [16].

Similarly, the court found claims relating to a combination of tramadol and acetaminophen in *Ortho-McNeil v. Teva* obvious [17]. Ortho claimed a tramadol and acetaminophen combination in U.S. Reissued Patent 39,221 (“the RE'221 patent”) [17]. A prior art U.S. Patent No. 3,652,589 (“the '589 patent”) disclosed a 1:10 weight ratio of tramadol to acetaminophen in a four compound combination whereas the RE'221 patent claimed a 1:7.1 weight ratio. The court ruled that the difference between the disclosed ratio and the claimed ratio was so slight that it created a presumption of obviousness [17]. This obviousness presumption was not rebutted because Ortho was unable to prove that “the claimed range achieves unexpected results relative to the prior range” [17]. Additionally, Ortho could not show that the prior art taught away from lowering the ratio of tramadol to acetaminophen from 1:10 to 1:7.1 [17].

### 3.2. Characteristics of a Drug Need Not Be Expressly Stated in the Prior Art in order to Be Used as Prior Art

The claims in U.S. Application No. 12/167,859 (“the '859 application”) and U.S. Application No. 11/766,740 (“the '740 application”) were deemed to be obvious in *In re Huai-Hung Kao* [18]. The claims at issue in the '859 application were for a method of treating pain by administering oxymorphone in a controlled release formulation that provides at least 12 hours of sustained pain relief and results in a maximum concentration of at least about 50% higher when administered to fed verses fasting patients [18]. Endo, the patentee, argued that the prior art did not expressly disclose the “food effect limitation” and the 12 hour effectiveness limitation so its claims were nonobvious [18]. The Board of Patent Appeals and Interferences and the court held that both limitations need not be expressly disclosed in order for the new claims to be obvious because the prior art disclosed that oxymorphone's dissolution rate is 60%–80% after 12 hours so oxymorphone must still be effective after 12 hours [18]. The court also noted that the “food effect” is an inherent property of oxymorphone present in both controlled release formulations and immediate release formulations [18].

The claims in the '740 application in *In re Huai-Hung Kao* claimed the same oral controlled release formulation–an extended release formulation with an average increased bioavailability of about 26% for subjects with renal impairment compared to healthy subjects [18]. The Federal Circuit affirmed the Board by indicating that informing someone of a correlation between, for example renal failure and bioavailability, does not confer patentability absent a functional relationship between the informing and administering [18].

The two patents, U.S. Patents 6,254,887 (“the '887 patent”) and 7,074,430 (“the '430 patent), in *Purdue v. Par* were affirmed by the Federal Circuit to be invalid for obviousness [19]. The district court held that the asserted claims for controlled release tramadol formulations suitable for once-daily oral dosing were obvious in light of prior art that disclosed formulations of opioid analgesics, including tramadol, and once-daily formulations [19]. Specifically, one prior art patent, U.S. Patent No. 5,580,578, expressly listed tramadol as one of fourteen different opioid analgesics for use in a controlled-release formulation that provided effective blood levels for 24 hours [19].

### 3.3. Claims Are Not Obvious when There Are Unexpected Properties

It would seem that there is little hope for nonobvious formulations. But all is not lost. Where the patentee can demonstrate that the formulation exhibits an unexpected property or where evidence shows that the art, as a whole, teaches away from a certain combination of elements, patent claims to formulations have been found to be nonobvious. The following cases illustrate this point.

#### 3.3.1. Unexpected Properties

In *Abbott v. Sandoz,* the Federal Circuit affirmed the district court's decision to enjoin Sandoz from making or selling an extended release formulation of clarithromycin given Abbott Laboratories' U.S. Patent Nos. 6,010,718 (“the '718 patent”) and 6,551,616 (“the '616 patent”) [20]. The '718 patent claimed an extended release pharmaceutical composition comprising an erythromycin derivative and a pharmaceutically acceptable polymer, whereby after ingestion certain specified parameters of drug bioavailability are met [20]. The '616 patent claimed a method of reducing gastrointestinal side effects [20]. Sandoz, the patent challenger, claimed that the extended release formulations of clarithromycin claims were obvious because the prior art disclosed all aspects of the claims. Specifically, PCT Patent Publication WO 95/30422 described a controlled-release dosage form and pharmacokinetic properties of azithromycin, the European Patent Publication No. 0,280,571 B1 disclosed the extended release formulations of erythromycin derivatives, and U.S. Patent No. 5,705,190 described the modified release alginate salt formation of clarithromycin [20]. Sandoz argued that Abbott merely “pursue[d] known options” from the prior art for both the '718 and '616 patents [20]. In response, Abbott indicated that its choice of extended release components was not shown or suggested by the prior art to produce the pharmacokinetic properties of its claims [20]. Abbott further showed, and the court agreed, that there were such significant differences among erythromycin derivatives azithromycin and clarithromycin in absorption, distribution, metabolism, and excretion profiles that a skilled artisan would not have known the effect of substituting clarithromycin for azithromycin in any specific formulation [20]. The district court also discussed that it was not predictable, from the in vitro behavior of azithromycin, how any specific clarithromycin extended release formulation would perform in vivo [20]. The district court concluded that the unexpected properties of the new controlled release formulation led the claims to be nonobviousness [20].

Unpredictable results also led the district court and the Federal Circuit in the *In re Omeprazole Patent Litigation* to conclude that claimed formulations containing omeprazole were nonobvious over the prior art [21]. The patents at issue, U.S. Patent Nos. 4,786,505 (“the '505 patent”) and 4,853,230 (“the '230 patent”), added an inert subcoating that rapidly disintegrates in water into the formulation so that there is an increase in storage stability in addition to a resistance to gastric acid to prevent omeprazole from degrading in the stomach [21]. Apotex, the patent challenger, argued that the claims in the '505 and '230 patents were obvious in light of the prior art disclosing tablet coatings [21]. In particular, a published European patent application described an omeprazole tablet with an enteric coating [21]. Apotex used the prior art disclosure of an enteric coated omeprazole tablet and references disclosing the use of subcoatings in various pharmaceutical preparations to support the idea that it would have been obvious to apply an inert subcoating to the omeprazole tablet disclosed in the European patent application [21]. The district court disagreed and found that a person of ordinary skill would not have had a reason to apply a subcoating to the tablets because the prior art does not disclose, suggest, or infer that there was a negative interaction between the drug core and the enteric coating to require a subcoating for the tablets [21]. Further, there was no disclosure in the European patent application that suggested any need to stabilize omeprazole beyond using the salt form nor was there evidence that an enteric coating would create a problem resulting from contact with omeprazole [21]. Thus, the district court concluded, as affirmed by the Federal Circuit, that a person of ordinary skill in the art would not have seen any need to apply subcoatings to the tablets disclosed in the European patent application [21].

#### 3.3.2. Teaching Away

The district court, affirmed by the Federal Circuit, held in *Unigene Labs v. Apotex*, that U.S. Patent No. RE40,812E (“the '812E patent) was not obvious at time of invention [22]. The '812E patent covered Fortical®, a bioequivalent form of Miacalcin® [22]. Although both Fortical® and Miacalcin® contained salmon calcitonin, the two have different formulations with Fortical® containing 20 mM of citric acid that functions as an absorption enhancer and stabilizer, and Miacalcin® containing 0.10 mg of benzalkonium chloride that functions as a preservative, absorption enhancer, and surfactant [22]. The '812E patent claimed salmon calcitonin containing 20 mM of citric acid as an absorption enhancer and stabilizer [22]. The district court found that no prior art taught using 20 mM citric acid to achieve “both shelf stability and enhanced bioavailability” in a nasal salmon calcitonin formulation and that it was not obvious to use citric acid to modify Miacalcin® [22]. The court further noted that although prior art U.S. Patent No. 5,912,014 (“the '014 patent”) disclosed the use of citric acid in a pharmaceutical formulation, a person of ordinary skill would not use 20 mM of citric acid in the normal course of research and development for two reasons: (1) citric acid is used in concentrations much higher than the 20 mM found in Fortical® in the '014 patent; and (2) the increased bioavailability demonstrated in the '014 patent was in the context of a liquid injection into a rat duodenum, and not demonstrated for human use in a liquid pharmaceutical formulation [22]. In coming to its conclusion, the court also relied on two other prior art references that disclosed citric acid as a pH adjuster or buffer rather than as an absorption enhance and stabilizer, noting that such disclosures taught away from using 20 mM citric acid as an absorption enhancing agent or stabilizing agent in a liquid formulation [22].

## 4. Thoughts about Formulation Patents

Obviousness is a question of law based on facts [18]. While the scope of patent claims may be vast and can encompass anything under the sun invented by man [23], the line around what will be found to be obvious seems to be drawn around formulations where the combination or the properties are either disclosed in the prior art or predictable from the prior art. Practically, this conclusion leads to a need to clearly show why the formulation is not obvious, such as the court did in *Abbott* [20].

As shown in the recital of recent cases involving formulation patents and obviousness, there are two ways to overcome a finding of obviousness. One way is for the patent applicant to demonstrate unexpected properties in the new claim in light of what is already known from the prior art. The second way is to demonstrate that there is prior art that teaches away from making the new claim. In short, experimental data about why or how a formulation behaves in some unexpected way can constitute powerful evidence that the claims to such a formulation are not obvious.

## Figures and Tables

**Table 1.** Orange book listings for clobetasol.

**Dosage Form**	**Patent No.**	**Patent Expiration**	**Independent Claim**
Aerosol, Foam	6,126,920	March 1, 2016	A method of treating a skin disease susceptible to treatment with corticosteroid active substances, said method comprising administering topically to a patient in need thereof, an effective amount of a foamable pharmaceutical composition comprising a corticosteroid active substance, a quick-break foaming agent that comprises an aliphatic alcohol, water, a fatty alcohol and a surface active agent; a propellant; and a buffering agent present in an amount sufficient to provide a pH within the range of 3.0 to 6.0.
Lotion	6,106,848	September 22, 2017	A stable, topically applicable oil-in-water emulsion which is topically applicable to skin having intermediate viscosity, comprising (a) from 30% to 50% by weight relative to the total weight of said emulsion of at least one glycol; (b) at least one emulsifying agent comprising an anionic amphiphilic polymer; and (c) at least one biologically active agent, wherein said anionic amphiphilic polymer is present in an amount which in the absence of another emulsifying agent results in an emulsion having an intermediate viscosity, wherein said intermediate viscosity is a viscosity which ranges from 3 to 10 Pa.multidot.s (3,000 to 10,000 centipoises), measured with a Brookfield viscometer LVDV II+paddle No. 4, at a speed of 30 revolutions/minutes for thirty seconds, and at a temperature of 25 °C ± 3 °C.
Shampoo	7,316,810	June 17, 2019	A foaming composition for washing and treating the hair and/or the scalp consisting of 0.24 g citric acid, 2.6 g sodium citrate, 2 g polyquaternium 10, 17 g of sodium lauryl ether sulphate (2 moles of ethylene oxide, comprising 70% of active material), 6 g of cocoyl betaine (comprising 32% active material), 0.05 g clobetasol propionate, 10 g ethanol (95/96%), and q.s. for 100 g purified water.
Spray	5,990,100	March 24, 2018	A pharmaceutical composition for use in the treatment of psoriasis comprising (a) 0.0001 to 30 weight percent of an anti-psoriatic agent selected from the group consisting of corticosteroids, calcipotriol, retinoids, tar, and mixtures thereof; and (b) 15 to 97 weight percent isopropyl myristate, said component (a) being present in an effective anti-psoriatic weight percent, said pharmaceutical composition being a liquid and being in a form suitable for topical administration to a human.
